# Supplementary material for: Testing predictive coding theories of autism spectrum disorder using models of active inference
Source: PLoS Comput Biol. 2023 Sep 11;19(9):e1011473. doi: 10.1371/journal.pcbi.1011473 (PMC10529610; doi:10.1371/journal.pcbi.1011473)
Supplement: Attachment — Submitted filename: response to reviewers.docx [file pcbi.1011473.s001.docx]

**Comments to the Authors**

**Reviewer #1**

This manuscript uses computational modelling (HGF and two associative learning models) to determine whether any of the three leading predictive processing accounts of autism are plausible. The study is well designed and easy to follow, with succinct explanations of the theories being compared. This topic is very important, and the study does a good job at distinguishing the theories computationally, and explaining the results. I am especially appreciative of the use of very specific and relevant behavioural data (including active components, which is essential to the theories of active inference, but often neglected) and would be interested to hear what the authors think about perhaps extending this analysis to similar neural signals in the future. Overall, the manuscript was thorough and clear.

- **Response:** Thank you for the positive comments about the manuscript.

I would like the authors to add some information about whether it is fair to compare models with different inputs (as in the felt and veridical data for the SWI experiment), and what it means for conscious perception vs. action that the preferred model is (maybe?) different between these two measures.

- **Response:** We intended the comparison of the ‘felt’ and ‘veridical’ models as a way of seeing if the true weights of objects or how heavy they felt was responsible for the grip forces. This was really intended as a sense check, because the literature shows that predictive grip forces are scaled to true weights rather than the illusory perceptual effects, so we assumed that true weights would be the better way to construct the models. We felt that by running both versions we could check that the veridical (rather than felt) inputs could better explain the fingertip forces. But on reflection, we agree that the direct comparisons of the two types is perhaps unfair because it is not truly the same dataset.

In light of this comment, we have removed the direct comparison of the ‘felt’ v ‘veridical’ models using the Bayesian model selection procedure (and now just compare within each type). We think, however, that the more qualitative comparison of the log model evidence between the two can still be informative for providing this sense check. Therefore, we have reframed this comparison in the paper slightly as checking our prior assumption that the true weights were the best way to construct the model. See changes 261-283.

Regarding the winning model for each type, it is now clear that the HGF4 was the best for both versions, which provides further evidence that an additional level was needed for this task.

I am concerned about the difference in sample size between groups for the second experiment, and do not see that this is addressed by the analysis. This may impact on the results, as Figure 5 suggests there is far greater variability in the neurotypical group? This should be addressed in the next version of the manuscript.

- **Response:** As now clarified in the manuscript, a large neurotypical sample was recruited in the original study to provide sufficient power for correlational analysis (e.g., as in our Supplementary Materials). Although medium-to-large between-group effects were expected in these previous *a-priori* calculations (*d* = .78), it was noted that relationships between autistic-like traits and sensorimotor control are generally more moderate in magnitude. Hence, it was deemed that an overall sample of ~72 participants would be required to detect medium-sized correlations (r = .32; *α* = .05; *1 – β* = 0.80) akin to those observed in previous studies (e.g., Arthur et al., 2019). The size of the control group was increased to provide a more representative characterisation of how autistic-like traits vary across the general population (Palmer et al., 2015), while maintaining a ratio that aligns with clinical research recommendations (i.e., between 1:2 and 1:4; see Kang et al., 2009). We have added some of this rationale to the *Participants* section (see Lines 383-387).

We were aware of the differences in variance when performing the statistical analysis and did consider the best analysis approach. Our understanding is that ANOVA is typically quite robust to deviations from normality or unequal variances (e.g., Norman, 2010), and if anything will be conservative in terms of significance. Detecting the interactions was an important part of the analysis and we are not aware of a non-parametric equivalent to ANOVA that would allow us to test the interaction in the same way. So we chose to stick with the traditional parametric test. For the follow-up t-tests we have, however, used Welch’s t-test when there were unequal variances. We have added a footnote to the paper with this explanation. If you feel this is not a valid approach we are willing to explore other ways of analysing the data, but it seems unlikely that it will have led to spurious effects, particularly given the strength of the effects shown in the non-parametric follow-up tests. We have added text in a footnote to describe our approach (P23)

I would appreciate some discussion about why the two experiments might have different winning models (HGF3 v 4), and why the 4-level model would be preferred to model the data with less temporal complexity. I do like the structure of the general discussion as it stands, so perhaps this could be included in the discussion for Expt 2.

- **Response:** In our discussion of Expt 1, we suggest that people’s beliefs *about volatility* may well have been unstable in the object lifting protocol, “given that participants did not know what kinds of objects to expect or in what order they might be received”. For Expt 2, we think that participants may have been less uncertain about the variety of trial outcomes and trial order changes. There were only two ‘types’ of ball in the racquetball experiment, and the likelihood of getting an unexpected one was changing quite regularly. Conversely, participants appeared to have high uncertainty about the number of different objects in Expt 1. It is typical that participants believe there were more different objects than there really were in these experiments which could account for the additional uncertainty that needed to be captured in the models. We have added some text to discuss this at lines 278-283 and 503-507.

I am unsure why the posthoc analysis was done for an insignificant interaction (Exp2 α2).

- **Response:** We ran these because even though the interaction was not statistically significant in our ANOVA, our plots strongly imply that some kind of interaction was present in the data. The follow up tests suggested that this was indeed the case. While this is not the traditional approach, we thought it was important to examine whether or not the groups diverged across stable/volatile conditions. We have added some additional text to the results to explain why we adopted this approach (lines 547-550).

I would appreciate a little more discussion and flagging that the volatility group differences for Expt 2 are the opposite of what Lawson et al. (2017)’s theory would predict. This would also be good to note in the abstract, as the results do not seem as clear as it suggests.

- **Response:** Thanks, yes we have reworked this section in the discussion (see Lines 633-650). While we did discuss this issue already, the way this section was organised made it seem as though the results were more supportive than they really were. We have also added a sentence to the abstract to specifically highlight that we didn’t find evidence that autistic participants were perceiving the tasks as more volatile.

I note a few small errors in the manuscript – pg. 15 references Figure 3E – I presume D is meant, as there is no E. Pg.19 says “Autistic Quotient questionnaire” this should read “Autism Spectrum Quotient”. Pg. 20 – I cannot see where the number of blocks is reported, please include this information.

- **Response:** Thank you for highlighting these errors – they have now been corrected.

**Reviewer #2**

***General Evaluation:*** This paper offers an engaging exploration of the predictive processing framework and its utility in understanding sensory and motor discrepancies in individuals with autism. By employing computational approaches to devise generative models, the authors successfully elucidate the system's underlying mechanisms, facilitating an enhanced interpretation of observed behaviours. The structure of the paper is logically organized, and the application of a robust methodology delivers noteworthy insights into the domain of clinical neuropsychology.

***Strengths:***
- The paper is logically structured, and the research question is unequivocally stated. The authors' detailed explanation of the predictive processing framework and its relevance to understanding autism is praiseworthy.
- The innovative application of computational models to uncover the system's underlying mechanisms adds a fresh perspective to the study, promoting a more profound understanding of the observed behaviours.
- The authors have applied two distinct datasets to investigate sensorimotor atypicalities in autism, bolstering the validity of their findings.
- The provision of data and code on the OSF platform enhances transparency and reproducibility.

***Areas for Improvement:***
The relationship between the results and Active Inference rather than Predictive Processing in a conventional sense is somewhat unclear. For instance, the models and conclusions seem to omit Free Energy minimization or explicit epistemic reward (e.g., lines 33-35; 191-194; 379-382; 388-391; 717).

- **Response:** Thanks, yes the accounts we discuss are not strictly active inference accounts, and were possibly not written with more overt (oculo)motor actions in mind, but the work in this paper is attempting to extend these ideas further into the domain of naturalistic action. Consequently, there are still questions about how the atypical processing described in these theories maps onto actions. We have added some text to this effect in the discussion (lines 658-664).

The authors model actions discretely. An explanation and the discussion about how to adopt a continuous approach (as opposed to a median split, lines 264-266) could enrich the paper (lines 724-725).

- **Response:** Thanks, we have added some additional explanation for this choice and discussion about this at lines 488-493.

Method: While the methodology section is thorough, it could benefit from a more explicit delineation of the various components in the models. This enhancement could make the study more comprehensible to readers less familiar with this modelling approach.

- **Response:** We assumed that this comment referred to the HGF models rather than the associative learning ones so have added some additional description (see lines 180, and 194-209). Hopefully this provides some more explicit description of the key parts of the model, but we are happy to add more if there is anything specific that has been missed.

Figure 5F depicts distributions that are non-Gaussian, suggesting the authors may need to employ non-parametric tests to compare Autism and Neurotypical subjects.

- **Response:** Thanks, yes the other reviewer also noted the differences in variance between the autism and neurotypical groups. ANOVA is typically quite robust to deviations from normality or unequal variances (e.g., Norman, 2010), and if anything will be conservative in terms of significance. So we chose to stick with the traditional parametric test for most of the omnibus comparisons. While for the follow-up t-tests we have, however, used Welch’s t-test. We have added text in a footnote to describe our approach (P23). However, as you note the distributions are visibly skewed with a large tail for the α_3_ parameter, so we have replaced the ANOVA with some non-parametric tests as suggested (lines 555-559).

The authors evaluate the statistical power required in both studies but do not specify whether the observed effect sizes align with these estimations.

- **Response:** We have added a couple of sentences to each of the methods sections where we talk about power to discuss the effect sizes that we did see (line 107 and 402).

Discussion: The discussion could delve more deeply into the implications of the findings for our understanding of autism and the predictive processing framework. Questions about the relationship with key diagnostic symptoms, particularly social aspects, and the clinical and general applicability of these findings, remain.

- **Response:** We agree that there are many more questions that remain in the field and now acknowledge this on line 655-664. Due to the scope/length of the manuscript, we are not able to fully delve into broader social and behavioural research. However, to recognise the growing interest of predictive processing theories, we have pointed readers to some relevant recent evidence and materials.

The perspectives section should consider referencing neuroimaging and how it could resolve remaining questions (lines 703-705).

- **Response:** As recommended, we now make reference to neuroimaging methodologies in our discussion, on page 29 (lines 643 and 655). But we do so only briefly, because the focus of this work was more on moving away from simple tasks in brain scanners towards more naturalistic movement tasks.

Figures: The use of bar plots (Fig. 2A & 4C) is not advised as they can be misleading for non-Gaussian distributions. Box plots (like in Fig. 3 & 5) or violin plots might be more appropriate.

- **Response:** We have changed these for 2A and 4C and just plotted the raw data points with means and 95%CIs. For 4D and 2C we have left them as bar plots because they are just singular values, wo don’t have any error bars/data points to plot.

The term "cyan" is used in Figure 4E, but "magenta" appears more accurate (both in the Figure itself, and its caption line 551).

- **Response:** Thanks, yes definitely not cyan. Changed to ‘pink’.

Language: Please take care with the terminology used when discussing the distinctive aspects of autism, specifically avoiding words like "aberrant" (abstract & line 74).

- **Response:** We have checked the paper and removed terminology like ‘aberrant’ or ‘impairment’ where possible. The only time that terms like this are now used is when we are describing specific conclusions or hypotheses that have been made in *previous* theories (by other researchers) that specifically refer to ‘aberrant encoding of precision’. Generally, our data provides evidence against these notions, which we explicitly clarify in our discussions.

**Further References:**

Arthur, T., Vine, S., Brosnan, M., & Buckingham, G. (2019). Exploring how material cues drive sensorimotor prediction across different levels of autistic-like traits. *Experimental Brain Research, 237,* 2255-2267.

Kang, M. S., Choi, S. H., & Koh, I. S. (2009). The effect of increasing control-to-case ratio on statistical power in a simulated case-control SNP association study. *Genomics & informatics, 7(3),* 148-151.

Palmer, C. J., Paton, B., Kirkovski, M., Enticott, P. G., & Hohwy, J. (2015). Context sensitivity in action decreases along the autism spectrum: a predictive processing perspective. *Proceedings of the Royal Society B: Biological Sciences, 282(1802),* 20141557.

Norman, G. (2010). Likert scales, levels of measurement and the “laws” of statistics. *Advances in health sciences education*, *15*, 625-632.
